# Cancer and Pregnancy: Update of Estimates in Italy by Linking Data from Cancer Registries and Hospital Discharge Records

**DOI:** 10.3390/cancers17071230

**Published:** 2025-04-05

**Authors:** Daniela Pierannunzio, Alice Maraschini, Tania Lopez, Serena Donati, Edoardo Corsi Decenti, Paola Ballotari, Francesca Bella, Fortunato Bianconi, Ettore Bidoli, Rossella Bruni, Claudia Cirilli, Rosa Pasqualina De Vincenzo, Giovanna Fantaci, Giuseppe Furgiuele, Silvia Iacovacci, Antonella Ippolito, Lucia Mangone, William Mantovani, Elisabetta Merlo, Michael Mian, Walter Mazzucco, Maria Teresa Pesce, Giuseppe Sampietro, Giovanni Scambia, Fabrizio Stracci, Antonina Torrisi, Maria Francesca Vitale, Manuel Zorzi, Silvia Francisci

**Affiliations:** 1 National Centre for Disease Prevention and Health Promotion, Italian National Institute of Health, 00162 Rome, Italy; daniela.pierannunzio@iss.it (D.P.); serena.donati@iss.it (S.D.); edoardo.corsi@iss.it (E.C.D.); silvia.francisci@iss.it (S.F.); 2 Technical-Scientific Statistical Service, Italian National Institute of Health, 00162 Rome, Italy; alice.maraschini@iss.it; 3 SC Osservatorio Epidemiologico—ATS Val Padana, 46100 Mantova, Italy; paola.ballotari@ats-valpadana.it; 4 Siracusa Cancer Registry, Provincial Health Authority of Siracusa, 96100 Siracusa, Italy; francesca.bella@asp.sr.it; 5 Area Operativa ICT-PuntoZero Scarl, 06128 Perugia, Italy; fortunato.bianconi@puntozeroscarl.it; 6 Cancer Epidemiology Unit, Centro di Riferimento Oncologico di Aviano (CRO) IRCCS, 33081 Aviano, Italy; bidolie@cro.it; 7 ARess Puglia, Registro Tumori, 70121 Bari, Italy; r.bruni@aress.regione.puglia.it; 8 Servizio di Epidemiologia e Comunicazione del Rischio, Dipartimento di Sanità Pubblica, Azienda Ausl di Modena, 41121 Modena, Italy; c.cirilli@ausl.mo.it; 9 Dipartimento Scienze Della Salute Della Donna, del Bambino e di Sanità Pubblica, Fondazione Policlinico Universitario Agostino Gemelli IRCCS, 00168 Rome, Italy; rosapasqualina.devincenzo@policlinicogemelli.it (R.P.D.V.);; 10 Diapartimento Ostetricia e Ginecologia, Università Cattolica del Sacro Cuore, 00168 Rome, Italy; 11 Registro Tumori di Trapani (ASP Trapani), 91100 Trapani, Italy; giovanna.fantaci@asptrapani.it; 12 Direzione Dipartimento di Prevenzione ASP Catanzaro, 88100 Catanzaro, Italy; giuseppefurgiuele@yahoo.it; 13 UOC Prevenzione Attiva ASL Latina, 04100 Latina, Italy; s.iacovacci@ausl.latina.it; 14 ASP Ragusa, Dipartimento Prevenzione, UOSD Registro Tumori, 97100 Ragusa, Italy; antonella.ippolito@asp.rg.it; 15 Azienda Unità Sanitaria Locale, IRCCS di Reggio Emilia, 42122 Reggio Emilia, Italy; lucia.mangone@ausl.re.it; 16 Clinical and Evaluative Epidemiology Unit, Healthcare Trust of Trento, 38123 Trento, Italy; william.mantovani@apss.tn.it; 17 SC Epidemiologia ATS Brianza, 20900 Monza, Italy; elisabetta.merlo@ats-brianza.it; 18 Innovation, Research and Teaching Service (SABES-ASDAA), Teaching Hospital of the Paracelsus Medical Private University (PMU), 39100 Bozen, Italy; michael.mian@sabes.it; 19 College of Health Care-Professions Claudiana, 39100 Bozen, Italy; 20 Clinical Epidemiology and Cancer Registry Unit, Palermo Province Cancer Registry, Palermo University Hospital “P. Giaccone”, 90127 Palermo, Italy; walter.mazzucco@unipa.it; 21 U.O.C Monitoraggio Ambientale e Registro Tumori (ASL Caserta), 81100 Caserta, Italy; mariateresa.pesce@aslcaserta.it; 22 Bergamo Cancer Registry (ATS Bergamo), 24121 Bergamo, Italy; giuseppe.sampietro@ats-bg.it; 23 Umbria Cancer Registry, 06128 Perugia, Italy; fabrizio.stracci@unipg.it; 24 Dipartimento Igiene e Medicina Preventiva, University of Perugia, 06100 Perugia, Italy; 25 Italian Association of Cancer Registries (AIRTum), 20148 Milano, Italy; 26 AOU Policlinico—San Marco Catania, UOC Igiene Ospedaliera—Registro Tumori Integrato Catania-Messina-Enna, 95123 Catania, Italy; torrisinina@gmail.com; 27 Napoli 3 Sud Cancer Registry, 80031 Brusciano, Italy; mafravi86@libero.it; 28 Registro Tumori del Veneto, Servizio Epidemiologico Regionale, Azienda Zero, 35131 Padova, Italy; manuel.zorzi@azero.veneto.it

**Keywords:** cancer, pregnancy, pregnancy outcome, reproductive health, childbearing age, record linkage, cohort study, population-based data, cancer registry records, hospital discharge data

## Abstract

**Background/Objectives**: The increasing incidence of cancer during pregnancy is a growing public health concern, driven by delayed parenthood and rising maternal age. Pregnancy-associated cancer (PAC) presents complex clinical challenges, necessitating a balance between maternal cancer treatment and fetal safety. Historically considered incompatible with favorable pregnancy outcomes, evidence now suggests that pregnancy can often proceed without affecting cancer prognosis. A 2022 study in Italy provided the first population-based PAC estimates by linking cancer registries (CRs) and hospital discharge records (HDRs). This study aimed to update PAC estimates to 2019, covering 30% of the Italian population and addressing prior data limitations. **Methods**: A retrospective longitudinal analysis was conducted on women aged 15–49 diagnosed with malignant cancers between 2003 and 2019. Data from 21 Italian CRs were linked with HDRs to identify PAC cases, defined as obstetric hospitalizations occurring for women diagnosed with cancer in our study cohort in the period spanning from one year before to two years after a cancer diagnosis. All malignant cancers, excluding non-melanoma skin cancers, were analyzed. PAC rates were calculated per 1000 pregnancies, and trends were assessed using log-linear and JoinPoint regression models. **Results**: Among 131,774 women diagnosed with cancer, 6329 PAC cases were identified, with a PAC rate of 1.43 per 1000 pregnancies, consistent with global estimates. Thyroid (24.4%) and breast cancer (23.2%) were the most common. Analyzing the PAC rate by pregnancy outcome, in the period 2015–2019, this increased for both childbirths and miscarriages but decreased for voluntary terminations. Most hospitalizations (54%) occurred pre-diagnosis, peaking at diagnosis, especially for breast cancer (69%). **Conclusions**: PAC incidence is rising, particularly for live births and miscarriages, underscoring the need for multidisciplinary care and robust epidemiological insights to guide clinical management.

## Simple Summary

Cancer during pregnancy is a rare event which is becoming more frequent, partly due to women having children later in life. Managing pregnancy-associated cancer (PAC) is complex, as doctors must treat the mother while ensuring the baby’s safety. Historically seen as incompatible, recent evidence shows that many pregnancies can continue without harming the mother’s cancer outcome. This study provides an updated comprehensive analysis of pregnancy-related complications in women diagnosed with cancer, exploring both time trends and the impact of cancer on reproductive outcomes. Among 131,774 cases, 6329 had hospital access due to pregnancy, corresponding to a PAC rate of 1.43 per 1000 pregnancies, consistent with global trends. Thyroid and breast cancers were the most common. The study also found that PAC rates increased for live births and miscarriages but decreased for abortions. The findings of this study are crucial for healthcare planning and improving care for women with cancer in their childbearing years, particularly in relation to obstetric and reproductive health.

## 1. Introduction

The coexistence of cancer diagnosis and pregnancy has attracted scientific interest over the past 20 years, fostering the proliferation of studies [1].

Over recent decades, there has been a gradual increase in the incidence of pregnancies complicated by pregnancy-associated cancer (PAC), defined as the occurrence of obstetric hospitalizations among women diagnosed with cancer in a period spanning from one year before to two years after a cancer diagnosis [2,3]. This trend primarily reflects higher maternal ages over time, but also depends on the underlying cancer incidence and changing patterns of birth rates in the general female population, as well as the improvements in cancer detection technology. Consequently, changes in any of these factors may impact PAC incidence trends. This is a possible reason why the increasing temporal trend is not recognized in all countries [4,5], and why it is paramount to have timely frequency data.

Several studies have aimed at quantifying PAC. Northern European countries, particularly Denmark [6] and Australia [7], have been pioneers. Over the past decade, Canada [8], the USA [9,10], and Italy [5,11,12] have also contributed significantly. Most studies converge on an estimate of approximately 1–3 cancer diagnoses per 1000 pregnancies [1]. When analyzing the phenomenon from the opposite perspective—counting the number of pregnancies within a cohort of women with cancer—recent estimates indicate that pregnancy occurs in 1% to 16% of cases, depending on age groups distribution within the cohort of women with cancer [10].

The most common cancer types in women with PAC mirror the frequency in the general population, with breast, thyroid, female genital organs, and melanoma being the most frequent PACs by type [12,13].

Although its frequency does not meet the official criteria of a rare disease, this unique situation raises similar issues, including the difficulty for clinicians to acquire enough experience in treating and the lack of evidence-based guidelines for all the different types of cancer encountered during pregnancy [14].

The challenges associated with clinical management have contributed to a proliferation of studies, particularly those examining the therapeutic implications related to the timing of cancer onset, whether during pregnancy or within 12 months postpartum. The diagnosis of an underlying malignancy during pregnancy may be delayed due to physiological changes associated with pregnancy which may alter pharmacokinetics and potentially influence the efficacy and safety of pharmacological treatments [15]. Early detection and treatment are needed not only for the mother’s health, but also for the safety of the fetus, raising ethical issues that both physicians and woman are often not ready to face [16].

A recent Swedish study reports a one-and-a-half-fold difference in the frequency of PAC between pre- and postpartum periods (28 vs. 73 per 100,000) [4]. The availability of different therapeutic options has even led clinicians to propose a revision of the PAC definition, for example, in the case of breast cancer to clearly distinguish between pre- and post-pregnancy cases [17].

The analysis of frequency becomes clinically significant as PAC can affect fetal outcomes, either by increasing the risk of cesarean deliveries and preterm births in women with PAC [13] or by contributing to adverse birth outcomes [18]. Similar results can be found in the Horizon Adolescent and Young Adult cohort of women with cancer, in USA [19].

In our previous study, deliveries were the most frequent pregnancy outcome among both women with PAC (68.9%) and the reference population (74.9%), identified as obstetric hospitalizations for all women in childbearing age. However, the ratios of voluntary termination of pregnancy (VTP) and miscarriage were significantly higher in women with PAC [20].

In general, there is a shortage of data on PAC frequency and trends, particularly in Southern Europe. This is mainly due to the scarcity of linkable population-based data on cancer and pregnancies.

The aim of this paper is to contribute to the understanding of PAC by addressing the main limitations of the previous study, particularly those related to data availability and updating. In the present study, a new data collection effort has extended the territorial coverage from 23% to 30% of the Italian population, and the cancer cohort has been updated by three years, extending it until 2019 and allowing the analysis of more recent years, possibly considering the rapid evolution of cancer therapies.

## 2. Materials and Methods

We conducted a longitudinal retrospective population-based study, examining women of childbearing age (15–49 years old) diagnosed with cancer. The cohort was identified using data from population-based cancer registries (CRs), which track incident cases of cancer diagnoses across different Italian territories and linking with hospital discharge records (HDRs), which track obstetric hospitalization.

After a first study conducted in 2018 with 19 CRs providing incident cases from 2003 to 2015 (22.6% of the Italian female population aged 15–49) [12], a new call was funded in 2020 to update the epidemiology of PACs. CRs that participated in the previous study added incident cases up to 2019 and new CRs also joined.

CRs collected detailed demographic and clinical data on cancer patients residing in the area covered by cancer registration, including age, gender, diagnosis date, vital status, cancer site, and morphology. The study focused on malignant cancers (International Classification of Diseases for Oncology, 3rd Edition—ICD-O-3) and used the earliest cancer diagnosis when multiple malignancies occurred; benign and uncertain cancers and non-melanoma skin types were excluded.

HDRs, which provide individual-level data on hospital discharges (including day-hospital and day-surgery), were analyzed to assess obstetric hospitalizations. These records contain demographic details, clinical diagnoses (including main and secondary discharge diagnoses), and procedure codes (including main and secondary interventions) using the International Classification of Diseases, 9th revision, Clinical Modification—ICD9-CM. Specific diagnostic and procedural codes were used to categorize obstetric outcomes, including miscarriage, VTP, ectopic pregnancy, hydatidiform mole, and birth (Appendix A). If an obstetric hospitalization could not be categorized into these outcomes, it was classified as “other”. If a woman experienced multiple hospitalizations resulting in one of the outcomes classified above, she was counted only once.

PAC cases were identified by selecting obstetric hospitalizations occurring between one year before and two years after cancer diagnosis [12]. To compute PAC rates (number of PAC per 1000 pregnancies), the number of pregnancies was obtained by obstetric hospitalizations for all women in childbearing age within the same reference territories as the participating cancer registries and during the same years of incidence.

*p*-values for testing the differences in the proportions of women with cancer and women with PAC in the distribution by cancer sites (testing the hypothesis of equal distribution in the two groups of population) were calculated by year of cancer incidence.

The analysis also investigated trends of PAC from 2003 to 2019, focusing on different pregnancy outcomes, using log-linear models. This approach allowed the researchers to identify annual percentage changes (APCs) and any statistically significant shifts in pregnancy-related outcomes over the study period. Significant changes in these trends were evaluated using the JoinPoint Regression Program with permutation testing; for all other analyses, SAS software version 9.4, 2013, was used.

The Istituto Superiore di Sanità (Italian National Institute of Health—ISS), in collaboration with the Italian Society of Gynecology and Obstetrics (SIGO) and the Italian Cancer Registries Association (AIRTUM), coordinated the study. The Ethics Committee of the ISS approved the protocol of the study (protocol code AOO-ISS 0028471—28 September 2018 and AOO-ISS 0000200—4 January 2022).

## 3. Results

Twenty CRs provided data for women diagnosed with cancers between 2003 and 2019, covering approximately 30% of the Italian female population aged 15–49 years old (from a minimum of 21% in 2003 to a maximum of 41% in 2013). They were distributed across Italy (nine from northern, two from central, and nine from southern Italy). Some CRs that previously covered a specific area are now regionally based.

Overall, around four million women of childbearing age were annually monitored from 2003 to 2019 by CRs. A detailed list of participating CRs and the year of incidence is provided in Appendix B.

From 2003 to 2019, a total of 131,774 women aged 15–49 years old were diagnosed with malignant cancers (excluding non-melanoma skin cancer) (Table 1), of which 4650 (3.5%) had multiple cancer diagnoses during the study period. During the same period, 4,432,102 pregnancies were recorded in the hospital discharge records for women aged 15–49. Additionally, 8256 obstetric hospitalizations occurred between one year before and two years after a cancer diagnosis, corresponding to 6329 women with PAC.

The overall PAC rate was 1.43 per 1000 pregnancies. The mean age of PAC increased over the analyzed period, rising from 33.6 years in 2003 to 34.9 years in 2019.

During 2003–2019, the most common cancer types among women of childbearing age with cancer were breast cancer (41.6%), thyroid and other endocrine gland cancers (15.4%), and cancers of the female genital organs (10.0%), with a median diagnosis age of 41 years old. For women with PAC, thyroid and other endocrine cancers were the most prevalent (24.4%), followed by breast cancers (23.2%), and melanoma of the skin (15.4%), with a median diagnosis age of 35 years. When comparing women with cancer and women with PAC, in the most frequent cancers, the difference between the two groups’ results were always significant (*p*-value < 0.05), except for female genital organs cancer (Table 2).

The PAC rate for women diagnosed with thyroid and other endocrine glands cancer was 0.35 per 1000 pregnancies in 2003–2019, rising from 0.28 in 2003 to 0.39 in 2019. The PAC rate for women diagnosed with breast cancer was 0.233 per 1000 pregnancies in 2003–2019, rising from 0.23 in 2003 to 0.59 in 2019.

**Figure 1:**
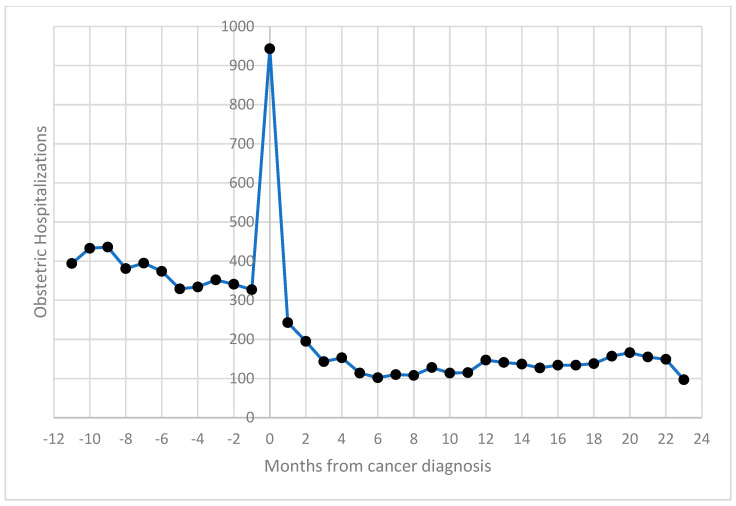
Distribution of obstetric hospitalizations of women aged 15–49 by distance (in months) from cancer diagnosis: 2003–2019, Italy.

Obstetric hospitalizations were more frequent before cancer diagnosis (54%), with a noticeable spike in admissions around the time of diagnosis (suggesting increased clinical monitoring during this period); 11.4% occurred within 30 days of the cancer diagnosis (Figure 1).

Throughout the entire period, the most common pregnancy outcome for all cancer types combined was childbirth (55%—slightly increasing compared to the period 2003–2015 when it was 53%), followed by miscarriage (12%—stable) and VTP (11%—decreasing compared to the 12% of 2003–2015) (Table 3). Childbirths after cancer diagnosis was approximately one third compared to the period before diagnosis.

VTP was most common output one year prior for all cancers, as well as for breast cancer, and one year after thyroid cancer diagnosis. For breast cancer, a significant decrease in all pregnancy outcomes was observed two years after diagnosis. Obstetric events increased when related to thyroid cancer diagnosis from the first to the second year after diagnosis.

Over 20% of obstetric hospitalizations were categorized as “other”; these admissions allow the identification of pregnancies with unknown outcomes.

**Figure 2:**
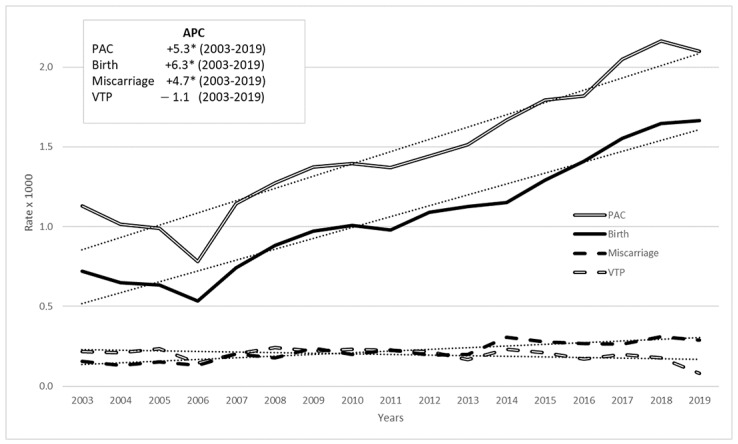
PAC rate (per 1000 pregnancies) time trend in 2003–2019 among women aged 15–49 by pregnancy outcome: all cancers, Italy, (annual percentage change—APC in box, linear trend in dots). * stands for statistically significant APC values (*p* < 0.05).

Analysis of trends in PAC outcomes from 2003 to 2019 revealed an increase in births and miscarriages, while VTP declined (Figure 2). These trends were confirmed by JoinPoint regression analysis: the APC for births was +6.3% from 2003 to 2019 (*p*-value < 0.05), and for miscarriages, it was +4.7% (*p*-value < 0.05), while for VTP there was a slight decrease (−1.1%) but the annual percentage change value was not statistically significant. The overall time trend for PAC outcomes showed a rise, with an annual percentage change of 5.3% (*p*-value < 0.05).

In the period 2003–2019, PAC and births continue to increase, albeit at a slower rate than in the period 2003–2015; miscarriages maintained growth but at a lower rate than PAC and births (in the period 2003–2015, they showed the highest growth rate).

From 2003 to 2019, the trend in PAC rate by maternal age sharply increased up to the 30–34 age group, remained stable for the 35–39 age group, and declined after that. For miscarriages and VTP, the trend increased up to the 35–39 age group, then decreased after that. This trend is unchanged from that of 2003–2015.

## 4. Discussion

Pregnancy-associated cancer is a highly complex occurrence, presenting significant challenges for both patients and healthcare providers.

Despite the need for epidemiological evidence to support clinical practice, uncertainties persist regarding the frequency and temporal trends of PAC.

Our study contributes to the field by providing updated estimates and confirmed trends regarding the rate of PAC in Italy.

The present study, following the same methodology already published and validated [12], included a greater number of years of cancer incidence data and a broader coverage of the Italian territory. The PAC rate estimate benefits from the inclusion of 80% additional women in the cohort, with cancer incidence data updated to 2019 and hospital discharge records extended to 2021.

The updated overall PAC rate of 1.43 per 1000 pregnancies shows a continued increase compared to the estimate of 1.24 reported in 2003–2015. Approximately 80% more women aged 15–49 diagnosed with malignant cancers were monitored in this study (the same increase was found in the number of women with PAC collected). This estimate aligns with the findings of a systematic review reporting an average of 1.09 PAC cases per 1000 pregnancies [13], and with other Italian studies reporting a rate of 1.34 per 1000 pregnancies [5,12]. The increasing incidence of PAC may be the consequence of: a. a shift in age of pregnancy toward ‘older’ ages, which implies higher cancer incidence; b. a change in diagnostic scrutiny and the use of increasingly sensitive diagnostic tests; c. increasing cancer incidence in childbearing age; d. increasing exposure or vulnerability to risk factors during pregnancy; or a combination of the above [21].

The trend in hospital admissions compared to the months from cancer diagnosis was comparable to that highlighted in the previous study [12].

Our research revealed a rise in PAC rates associated with birth and miscarriage (statistically significant), while rates linked to VTP showed a decline, though the change is not statistically significant. This trend may reflect the evolving approach to managing pregnancy alongside oncological treatment, highlighting that VTP may no longer be considered the sole option in these cases. Following the outpatient and day-service management of miscarriages, it is difficult to collect complete data on their trends in Italy. Therefore, although the hypothesis of a relative increase in miscarriages following the downward trend in VTP is plausible, we have no evidence to support it.

According to data from the International Network on Cancer, Infertility, and Pregnancy (INCIP) Registry, iatrogenic preterm birth caused by maternal oncological conditions, rather than chemotherapy exposure, was the leading cause of early postnatal complications and poor neonatal neurodevelopmental outcomes [22].

In a recent survey addressed to women with PAC, the analysis of responses showed an evolution in the types of questions posed to physicians, indicating that treatment is increasingly initiated during pregnancy. Specifically, it is observed that there was a rise in inquiries regarding treatment options and the timing of delivery, alongside a decline in questions concerning pregnancy termination [14].

PAC was most associated with breast, thyroid, and other endocrine cancers. The higher incidence of thyroid cancer among women with PAC compared with the previous data collection may be due to the greater diagnostic screening in Italy, which continues to increase [23], resulting in overdiagnosis. With respect to melanoma of the skin, both increasing incidence due to previous exposures and increasing diagnostic scrutiny could contribute to the high observed incidence. Differences in the ranking of cancers between the general female population aged 15–49 and women with PAC may be partly due to increased diagnostic scrutiny during pregnancy.

The study confirmed a similar distribution of obstetric hospitalizations relative to the timing of cancer diagnosis, with a peak in obstetric discharges occurring around the time of diagnosis. This trend likely reflects increased clinical monitoring for pregnant women diagnosed with cancer. Obstetric events occurring two years after cancer diagnosis were less frequent, likely due to women’s reluctance to conceive during or shortly after cancer treatments. This period warrants further research into fertility outcomes, since exploring the relationship between pregnancy outcomes, cancer types, and the interval since diagnosis could offer valuable insights for clinicians [24,25].

Trends by age group remain unchanged compared to the period 2003–2015; also, the distribution of PAC by outcomes across different age groups remained consistent with previous years, showing no significant shifts in the patterns observed in the earlier timeframe.

The first strength of our study is the population-wide coverage of cancer registries, which continues to increase and ensures that our results are representative of the entire population rather than a clinically selected cohort.

Another strength is the use of CRs to identify cancer cases. Cancer registries provide a high degree of accuracy and completeness in case identification, as well as detailed and reliable clinical information. This approach helps to overcome the methodological limitations associated with using only administrative health data, as is often the case with hospital discharge databases [26].

The first limitation of our study concerns the availability and timeliness of data: hospital discharge records are considered reliable and accessible at the national level only from 2003 onwards, and cancer registries gather incidence data retrospectively, typically with an average delay of three years. The study cohort is not fully representative of Italy, but it covers a significant portion of the national population—about 30%.

Additionally, obstetric events that do not result in hospitalization (such as early miscarriages) were not included in identifying pregnancies among women with cancer, leading to a potential underreporting of PAC in our results. However, the impact of this limit appears minimal, as our estimates are consistent with those found in other studies that used pregnancy surveillance data.

Obstetric hospitalizations were used to estimate pregnancy outcomes, with the limitation that home births were not included, as they are not recorded in hospital discharge records. However, home births represent a small proportion of the total births (the National Birth Register reported 0.1% between 2006 and 2019), and the study considers this negligible in terms of underestimating PAC occurrences.

The limitation due to the absence of high-resolution clinical variables, such as the TNM stage, which are not routinely collected by cancer registries has not yet been addressed. Information on the type and timing of treatment is also lacking, which might have relevant side effects and might impact pregnancy outcomes. In future work, it would be valuable to examine PAC by clinical outcome and stage at diagnosis. Furthermore, for future research, the study design and methodology proposed in this study are applicable to other countries if cancer registries and hospital discharge records are available.

The integration of information on pregnancy, delivery, gestational age, and parental characteristics from the Certificate of Delivery Assistance registry (CEDAP) could provide valuable evidence to inform clinical practice [27]. To this purpose, the feasibility of utilizing cancer registry data linked to CEDAP in the Veneto Region is under investigation. Further research will be conducted to thoroughly assess the factors influencing maternal survival rates and infant mortality, with the aim of gaining deeper insights into these critical health issues [18,28].

## 5. Conclusions

The findings from this study provide valuable insights into the intersection of cancer diagnoses and pregnancy outcomes among women of childbearing age. By leveraging a large, population-based dataset, it contributes to understanding how cancer and its treatment may influence reproductive health, specifically the occurrence of PACs in Italy. The detailed examination of obstetric hospitalizations around the time of cancer diagnosis also highlights the potential risks and challenges faced by women in this cohort. The study’s findings are essential for informing public health policies and cancer control programs, particularly those aimed at supporting women who are diagnosed with cancer during their reproductive years.

This study provided a comprehensive analysis of pregnancy-related complications in women diagnosed with cancer, exploring both time trends and the impact of cancer on reproductive outcomes. Its findings are crucial for healthcare planning and improving care for women with cancer in their childbearing years, particularly in relation to obstetric and reproductive health.

## Figures and Tables

**Table 1:** Distribution of women with cancer diagnosis, number of pregnancies, number of women with PAC, rate of PAC (per 1000 pregnancies), and mean age of PAC by year of cancer incidence: ages 15–49, all cancers, 2003–2019, Italy.

Year of Cancer Incidence	Women with Cancer Diagnosis	Pregnancies	PAC	PAC Rate per 1000	Mean Age of PAC
2003	4828	238,151	269	1.13	33.6
2004	5048	247,262	251	1.02	34.0
2005	5252	253,467	251	0.99	34.5
2006	5449	303,968	238	0.78	34.5
2007	6396	282,653	323	1.14	34.6
2008	7453	294,341	375	1.27	34.7
2009	8046	298,538	410	1.37	34.4
2010	8031	294,566	411	1.40	34.6
2011	7914	279,843	383	1.37	34.4
2012	7883	273,134	394	1.44	33.9
2013	11,163	312,163	472	1.51	35.0
2014	10,981	294,846	492	1.67	34.7
2015	10,660	274,536	492	1.79	34.2
2016	10,445	267,301	486	1.82	34.6
2017	10,262	259,235	531	2.05	35.1
2018	6680	147,026	318	2.16	34.9
2019	5283	111,072	233	2.10	34.9
2003–2019	131,774	4,432,102	6329	1.43	34.5

**Table 2:** Distribution of women aged 15–49 and women with PAC aged 15–49 by cancer type (absolute and percent values): 2003–2019, Italy.

Topography * (ICDO-3)	Women 15–49 with Cancer Diagnosis		Women with PAC 15–49		*p*-Value
	N	%	N	%	
Breast (C50)	54,841	41.62	1466	23.16	0.000
Thyroid and other endocrine glands (C73–C75)	20,287	15.40	1543	24.38	0.000
Femal genital organs (C51-C58)	13,126	9.96	613	9.69	0.465
Melanoma of the skin (C44 with morphology codes: 8720–8790)	11,098	8.42	977	15.44	0.000
Digestive organs (C15–C26)	9781	7.42	356	5.62	0.000
Lymph nodes (C77)	5413	4.11	363	5.74	0.000
Hematopoietic and reticuloendothelial systems (C42)	4765	3.62	305	4.82	0.000
Respiratory system and intrathoracic organs (C30–C39)	3883	2.95	174	2.75	0.345
Eye, brain, and other parts of central nervous system (C69–C72)	2431	1.84	172	2.72	0.000
Urinary tract (C64–C68)	2284	1.73	138	2.18	0.009
Lip, oral cavity, and pharynx (C00–C14)	1506	1.14	80	1.26	0.340
Connective, subcutaneous, and other soft tissues (C49)	889	0.67	65	1.03	0.000
Bones, joints, and articular cartilage of other and unspecified sites (C40–C41)	520	0.39	29	0.46	0.439
Unknown primary site (C80)	470	0.36	23	0.36	0.939
Retroperitoneum and peritoneum (C48)	236	0.18	13	0.21	0.631
Other and ill-defined sites (C76)	182	0.14	7	0.11	0.562
Peripheral nerves and autonomic nervous system (C47)	62	0.05	5	0.08	0.260
Total	**131,774**	**100.00**	**6329**	**100.00**	

* All morphologies were considered except for the skin (C44), where only the codes 8720–8790 were included.

**Table 3:** Distribution of women aged 15–49 with PAC by pregnancy outcome and timing (one year before cancer diagnosis, one year after cancer diagnosis, and two years after cancer diagnosis) for all cancers, breast cancers, and thyroid and endocrine glands cancer (counts, row %): 2003–2019, Italy.

**All Cancers**								
**Pregnancy Outcome**	**One Year Before Cancer Diagnosis**		**One Year After Cancer Diagnosis**		**Two Years After Cancer Diagnosis**		**Total by Pregnancy Outcome**	
	**N**	**%**	**N**	**%**	**N**	**%**	**N**	**%**
Birth	2694	59.2	925	20.3	934	20.5	4553	55.1
Miscarriage	559	58.2	192	20.0	209	21.8	960	11.6
Voluntary termination of pregnancy	417	45.9	318	35.0	174	19.1	909	11.0
Ectopic pregnancy and hydatidiform mole	68	54.0	33	26.2	25	19.8	126	1.5
Other	720	42.2	657	38.5	331	19.4	1708	20.7
**Total by time of diagnosis**	**4458**	54.0	**2125**	25.7	**1673**	20.3	**8256**	**100.0**
**Breast Cancer**								
**Pregnancy Outcome**	**One Year Before Cancer Diagnosis**		**One Year After Cancer Diagnosis**		**Two Years After Cancer Diagnosis**		**Total by Pregnancy Outcome**	
	**N**	**%**	**N**	**%**	**N**	**%**	**N**	**%**
Birth	699	74.3	210	22.3	32	3.4	941	53.1
Miscarriage	209	77.7	37	13.8	23	8.6	269	15.2
Voluntary termination of pregnancy	149	58.2	84	32.8	23	9.0	256	14.4
Ectopic pregnancy and hydatidiform mole	17	68.0	7	28.0	1	4.0	25	1.4
Other	126	44.8	125	44.5	30	10.7	281	15.9
**Total by time of diagnosis**	**1200**	67.7	**463**	26.1	**109**	6.2	**1772**	**100.0**
**Thyroid and Other Endocrine Glands Cancer**								
**Pregnancy Outcome**	**One Year Before Cancer Diagnosis**		**One Year After Cancer Diagnosis**		**Two Years After Cancer Diagnosis**		**Total by Pregnancy Outcome**	
	**N**	**%**	**N**	**%**	**N**	**%**	**N**	**%**
Birth	642	54.6	173	14.7	361	30.7	1176	59.3
Miscarriage	104	44.8	61	26.3	67	28.9	232	11.7
Voluntary termination of pregnancy	83	34.3	93	38.4	66	27.3	242	12.2
Ectopic pregnancy and hydatidiform mole	9	40.9	2	9.1	11	50.0	22	1.1
Other	141	45.2	66	21.2	105	33.7	312	15.7
**Total by time of diagnosis**	**979**	49.3	**395**	19.9	**610**	30.7	**1984**	**100.0**

## Data Availability

Data are unavailable due to privacy restrictions.

## Appendix A

**Table A1:** Codes for obstetric hospitalizations selection.

Hospital Discharge Records Are Selected If They Contain at Least One of the Following Criteria:		
Condition	Code	System
Complications of pregnancy, childbirth, and the puerperium	630–677	ICD9-CM principal or secondary diagnosis
Use of health services for pregnancy	V22, V23, V24, V27, V28	ICD9-CM principal or secondary diagnosis
Livebirth or stillbirth (specific neonatal code sometimes found in the woman’s HDR, neonatal records were excluded by age selection)	V30–V39	ICD9-CM principal or secondary diagnosis
Obstetrical procedures	72–75	ICD9-CM principal or secondary procedure
Obstetrical hospitalization	370–384	Diagnosis Related Groups (DRG)
Dilation and curettage for termination of pregnancy	69.01	ICD9-CM principal or secondary procedure
Dilation and curettage following delivery or abortion	69.02	ICD9-CM principal or secondary procedure
Aspiration curettage of uterus for termination of pregnancy	69.51	ICD9-CM principal or secondary procedure
Aspiration curettage following delivery or abortion	69.52	ICD9-CM principal or secondary procedure
Salpingectomy with removal of tubal pregnancy	66.62	ICD9-CM principal or secondary procedure

## Appendix B

Cancer Registry of Alto Adige (2003–2019)
Cancer Registry of ATS Brianza (2007–2015)
Cancer Registry of Mantova–Cremona (2003–2018)
Cancer Registry of Bergamo (2007–2017)
Cancer Registry of Veneto (2003–2019)
Cancer Registry of Modena (2018–2019)
Cancer Registry of ASL Napoli 3 Sud (2003–2017)
Cancer Registry of Catanzaro (2006–2010)
Cancer Registry of Messina–Catania–Enna (2003–2018)
Cancer Registry of Siracusa (2003–2018)
Cancer Registry of Palermo (2003–2019)
Cancer Registry of Trapani (2003–2014)
Cancer Registry of Ragusa–Caltanissetta (2003–2017)
Cancer Registry of Latina (2009–2013)
Cancer Registry of Umbria (2003–2017)
Cancer Registry of Emilia Romagna (2003–2017)
Cancer Registry of Caserta (2008–2017)
Cancer Registry of Trento (2003–2017)
Cancer Registry of Friuli Venezia Giulia (2003–2019)
Cancer Registry of Puglia (2013–2019)
